# Abnormal expression, localization and interaction of canonical transient receptor potential ion channels in human breast cancer cell lines and tissues: a potential target for breast cancer diagnosis and therapy

**DOI:** 10.1186/1475-2867-9-23

**Published:** 2009-08-18

**Authors:** Ebru Aydar, Syn Yeo, Mustafa Djamgoz, Christopher Palmer

**Affiliations:** 1 London Metropolitan University, Institute for Health Research and Policy, 166-220 Holloway Road, London, N7 8DB, UK; 2 Imperial College London, Division of Cell and Molecular Biology, Faculty of Natural Sciences, Sir Alexander Fleming Building, Exhibition Road, South Kensington, London, SW7 2AZ, UK

## Abstract

**Background:**

Ca^2+ ^is known to be involved in a number of metastatic processes including motility and proliferation which can result in store-depletion of Ca^2+^. Up regulation of genes which contribute to store operated channel (SOC) activity may plausibly be necessary for these processes to take place efficiently. TRPC proteins constitute a family of conserved Ca^2+^-permeable channels that have been shown to contribute to SOC activity.

**Results:**

In breast cancer biopsy tissues, TRPC3 and TRPC6 were the predominant TRPC genes expressed with TRPC3 and TRPC6 being significantly up regulated compared to normal breast tissue. In the lowly metastatic breast cancer cell line MCF-7, TRPC6 was the chief TRPC gene expressed while in the highly metastatic breast cancer cell line MDA-MB-231 both TRPC3 and TRPC6 were the predominant TRPC genes expressed. Western blotting, immunoconfocal analysis and immunoprecipitation experiments confirmed that the MDA-MB-231 cell line expressed both TRPC3 and TRPC6 protein with the majority of protein being intracellular. TRPC3 and TRPC6 were found to be in an immunoprecipitatble complex and co-localize within the cell. To demonstrate the potential of targeting TRP channels in breast cancer, hyperforin reportably a specific activator of TRPC6 significantly reduced the growth and viability of the breast cancer cell lines but had no effect on the non-cancerous breast cell line. Silencing of TRPC6 in MDA-MB-231 cells resulted in a significant reduction in cell growth but not viability.

**Conclusion:**

TRPC channels may be potential future targets for breast cancer diagnosis and therapy and deserve further investigation to evaluate their role in cancer cell physiology.

## Introduction

Breast cancer is a leading cause of cancer-associated death in women [1]. It most commonly metastasizes to the bone, with 70% of patients who develop bone metastases dying [2]. Finding early markers of metastasis and developing effective therapies against their development is a priority. Investigation of functional expression of membrane ion channels is an exciting development in cancer research. Increased expression of voltage-sensitive ion channels is directly associated with malignancy, as evidenced by their role in cell proliferation, migration and survival [3]. As such, some of these channels have begun to be developed as targets for cancer drug design [4]. Whilst considerable work has so far been done on K^+ ^and Na^+ ^channels, surprisingly little is known about the role of mechanosensitive channels and Ca^2+ ^signalling in cancer. Both are likely to have a significant role in the cancer process and Ca^2+ ^is a regulator of proliferation and apoptosis [5]. In this regard, a key group of ion channels of increasing prominence are the Transient Receptor Potential (TRP) family [6]. There is evidence that expression of TRPV6, TRPM8, TRPM1 and TRPV1 are significantly altered in human cancer cells [6]. TRPC proteins in humans (TRPC1, 3, 4, 5, 6 and 7) constitute a family of conserved Ca^2+^-permeable cation channels which are activated in response to agonist-stimulated PIP(2) hydrolysis and subsequent studies have provided substantial evidence that some TRPCs contribute to SOC activity [7]. TRPC proteins have also been shown to form agonist-stimulated calcium entry channels that are not store-operated but are likely regulated by PIP(2) or possibly diacylglycerol [7]. TRPC1 and 6 are also sensors of mechanically and osmotically induced membrane stretch [8]. In human PCa LNCaP cells, agonist-mediated stimulation of alpha1-adrenergic receptors required coupled activation of TRPC6 channels and nuclear factor of activated T-cells to promote proliferation [9]. Additionally, in the same cells, store-operated Ca^2+ ^channels were found to be important determinants of the transition to androgen-independent state [10]. Consistent expression of TRPC1, 3, 5, 6 in glioma cell lines and acute patient-derived tissues has been described. These channels gave rise to small, non-voltage-dependent cation currents that were blocked by the non-selective TRPC inhibitors GdCl_3_, 2-APB, or SKF96365. Importantly, TRPC channels contributed to the resting conductance of glioma cells and their acute pharmacological inhibition caused an ~10 mV hyperpolarization of the cells' resting potential. Additionally, chronic application of the TRPC inhibitor SKF96365 caused near complete growth arrest [11]. Finally in liver tumor samples TRPC6 was expressed more strongly than in isolated hepatocytes from healthy patients and experiments suggested that TRPC6 may play a role in control of human hepatoma cell proliferation [12]. Little is known about canonical TRP (TRPC) ion channels in breast cancer apart from a few emerging papers to date [13,14], such channels could play a major role in cellular activities involved in the cancer process since TRPC channels are predominantly Ca^2+ ^permeable. The primary aim of this paper is to determine which TRPC channels are expressed in breast cancer cell lines and tissues and the roles these channels may play in cell proliferation.

## Experimental details

### Materials

A mouse anti-TRPC3 polyclonal antibody was obtained from the Abnova Corporation and used at a 1:200 dilution for immunoblotting and 1:20 for immunochemistry. A rabbit anti-TRPC6 polyclonal antibody was obtained from Sigma-Aldrich and used at a 1:200 dilution for immunoblotting and 1:20 for immunochemistry. TBST buffer (10 mM Tris-HCl, pH 7.5, 150 mM NaCl, 0.05% Tween-20). PBS buffer (40 mM Na_2_HPO_4_, 10 mM KH_2_PO_4_, and 120 mM NaCl with a resulting pH of 7.2).

### Culture of cell lines

Human breast cancer cell lines (MCF-7 and MDA-MB-231) were cultured in DMEM (Gibco) containing 10% FBS and 4 mM glutamine with penicillin-streptomycin. The 'normal' human breast epithelial cell line (MCF-10A) was cultured in a 1:1 mixture of Dulbecco's modified Eagle's medium and F12 medium (Gibco, DMEM-F12) supplemented with 5% FBS, hydrocortisone (0.5 μg/ml), insulin (10 μg/ml), epidermal growth factor (20 ng/ml), and penicillin-streptomycin. All cell lines were maintained in a 37°C CO_2 _incubator in 100 mm culture dishes.

### RNA extraction from biopsy samples and breast cell lines

An absolutely RNA miniprep kit (Stratagene) was used to extract total RNA from breast cell lines or homogenized breast biopsy tissue. cDNA synthesis was conducted using 1 μg of RNA, 1 μg of Oligo (dT)_18 _and Superscript II reverse transcriptase (Gibco) according to the manufacturers instructions. All samples were tested for absence of genomic DNA contamination before use.

### PCR

Standard PCR reactions were performed with a Hotstar Taq plus PCR mastermix (Qiagen) under the following conditions: 95°C for 30 s, 55°C for 15 s, and 72°C for 1 min for 40 cycles. Heating for 5 mins at 95°C preceded the cycles. The primer concentration used was 0.5 μM. All primers were tested on known targets before use (refer to table 1). Nested PCR was used in the analysis of breast biopsy tissue. A standard PCR was performed using "external primers" as described above for 35 cycles. The "external primers" were designed approximately 100 bp upsteam/downstream of the "internal primers" following this step 1 ul of this PCR reaction was utilized in a new PCR reaction with "internal primers" in a 100 μl volume for a further 40 cycles.

**Table 1:** Primers utilized in this investigation

**Primer name**	**Primer sequence**	**Expected product size (bp)**
TRPC1 F	5'-GTA AGT GGA TTT GCT CTC AT-3'	298 for TRPC1B splice variant
TRPC1 R	5'-TGG TTA ATT TCT TGG ATA AA-3'	

TRPC3 F	5'-TAC TCA ACA TGC TAA TTG CTA TGA T-3'	383
TRPC3 R	5'-CAC AGT TGC TTG GCT CTT GTC TTC C-3'	

TRPC4 F	5'-CTC TGG TTG TTC TAC TCA ACA TG-3'	781
TRPC4 R	5'-CCT GTT GAC GAG CAA CTT CTT CT-3'	

TRPC5 F	5'-GCT CGC AGC CAC CCC AAA GGG AGG A-3'	548
TRPC5 R	5'-CCA ATG TCC CTA CCC TGT TCT CCC AGC TCT C-3'	

TRPC6 F	5'-GAA CTT AGC AAT GAA CTG GCA GT-3'	625 or 277
TRPC6 R	5'-CAT ATC ATG CCT ATT ACC CAG GA-3'	

TRPC7 F	5'-GTC CGA ATG CAA GGA AAT CT-3'	477
TRPC7 R	5'-TGG GTT GTA TTT GGC ACC TC-3'	

TRPM7 F	5'-AAA CCA GTT CTG CCT CCT CCA C-3'	100
TRPM7 R	5'-GTC CAT CGG AAG TCT TAT CTT TCT TTC-3'	

TRPM8 F	5'-GAT TTT CAC CAA TGA CCG CCG-3'	502
TRPM8 R	5'-CCCCAGCAGCATTGATGTCG-3'	

TRPV6 F	5'-GAG CAG TCC CTG CTG GAA CT-3'	254
TRPV6 R	5'-GGT ACT TCG AGA CAC TGA GG-3'	

Cav3.2 F	5'-GAA GGA CAC GCT GCG CGA GT-3'	180
Cav3.2 R	5'-GCA TCC TCC CGT GCC TCC TT-3'	

β-actin F	5'-ATG GAT GAT GAT ATC GCC GC-3'	350
β-actin R	5'-ATC TTC TCG CGG TTG GCC TT-3'	

TRPC1 F nested	5'-ATGGCGGCCCTGTACCCGAG-3'	
TRPC1 R nested	5'-AACACAATGTGCATTCACAG-3'	

TRPC3 F nested	5'-AAGTGACTTCCGTTGTGCTC-3'	
TRPC3 R nested	5'-AGGTTGCTGCATCATTCACA-3'	

TRPC4 F nested	5'-TGTGACCAATGTCAAAGCAC-3'	
TRPC4 R nested	5'-CTAACACACATTGTTCACTG-3'	

TRPC5 F nested	5'-CACATCACAACCACGACG-3'	
TRPC5 R nested	5'-ACTTGCCTGTAACAGATCGG-3'	

TRPC6 F nested	5'-CAA CCA GAA ACA GAA GCA TG-3'	
TRPC6 R nested	5'-CTC GCA ATG AAT GAT GCT GC-3'	

TRPC7 F nested	5'-CACAGTTCTCCTGGACAGAA-3'	
TRPC7 R nested	5'-TTACTTCAGATAAGCCGAAT-3'	

### Real time PCR

Real time PCR was performed on a DNA Engine Opticon 2 machine with Opticon Monitor™ software. PCR with SYBR^® ^Green PCR Master Mix (Qiagen) was performed under the following conditions: 95°C for 30 s, 55°C for 15 s, and 72°C for 1 min for 40 cycles. Heating for 5 min at 95°C preceded the cycles. Melting curves and agarose gel electrophoresis were performed to verify the specificity of the products. As variations in cDNA quality within different samples could occur, β-actin was included as an internal control. By subtracting the β-actin threshold (*C*_t_) value from the TRPC *C*_t _value of each sample, the cDNA quality of each sample is taken into account. The relative quantification (*R*) of the mRNA levels in each individual sample was calculated according to following equation: *R *= *E*^ΔCt (control-sample) ^[15]. The real time PCR efficiency of each TRPC amplicon (*E*) was calculated according to the method described previously [16].

### Protein methods

Cellular protein extracts at a concentration of 1 mg/mL were mixed with SDS sample buffer (Sigma-Aldrich) and 5 μg of protein were loaded per lane and separated on acrylamide 4% – 20% gradient Tris-glycine minigels (Cambrex). The proteins were transferred to nitrocellulose membrane for 3 h at 4°C; transfer was verified using Ponceau red (Sigma) and the blots were blocked with a solution containing 2.5% skimmed milk and 2.5% bovine serum albumin (BSA) in TBST overnight at 4°C before probing with specific antibodies. The antibodies were diluted in TBST with 0.5% skimmed milk and 0.5% BSA and incubated with the blot for 4 h at room temperature. Following four 10-min washes with TBST (with constant agitation) at room temperature, the blots were incubated with anti-mouse or anti-rabbit horseradish peroxidase-conjugated secondary antibodies (DAKO- at the manufacturers' suggested concentrations) as appropriate, and diluted in TBST with 0.5% skimmed milk and 0.5% BSA for 2 h at room temperature. The blots were again washed as before and then developed with an enhanced chemiluminescence Western blot kit (Amersham). Immunoprecipitations were carried out with a Classic Seize kit from Pierce. As controls, the immunoprecipitation was performed in the absence of immunoprecipitating antibody or with a isotype control antibody.

### Immunocytochemistry

Sterile 13 mm diameter glass coverslips were placed in 24 well costar plates. To each well 1 ml of cell suspension were seeded at a density of 2 × 10^4 ^cells/ml and then allowed to grow for 48 h. The cells were washed twice with PBS and then incubated with concanavalin-A (FITC conjugated) for 10 mins at room temperature in PBS with 1% BSA. Cells were washed twice in PBS for 1 min and then fixed in 2% paraformaldehyde for 7 mins at room temperature. The cells were washed four times with PBS and subsequently incubated in saponin (0.1%) in PBS for 5 mins at room temperature. The permeabilization solution was aspirated, and the cells were washed four times in PBS before being blocked with 8% BSA in PBS for 30 mins. Cells were again washed twice with PBS for 5 min and excess liquid was removed before addition of TRPC antibodies (1/20 dilution) in 1% BSA in PBS for 1 h. A further four PBS washes were performed before the cells were incubated with either an alexifluor 568 conjugated anti-rabbit IgG (1:100) or a FITC conjugated anti-mouse IgG (1:100) in PBS with 1% BSA for 30 mins at room temperature in the dark. Four 5 min PBS washes were performed in the dark and coverslips were mounted in Vectashield (Vector Laboratories Inc.) and allowed to set overnight at 4°C. Controls to assess auto-fluorescence or non-specific labeling were also performed and consisted of treatment with 1% BSA without either primary antibody, secondary antibody or both. Confocal microscopy was performed on a Leica 650T instrument using 568 and 488 nm filters and analysis performed with Leica confocal software using a cross section tool. Images were analyzed with excitation at both 568 and 488 nm and each singly to rule out 'cross' bleed from either wavelength.

### Silencing of TRPC6

Transfection was accomplished using a double transfection procedure using the following target sequences for TRPC6 (SiRNA1: AACGAGAGCCAGGACTATCTG; SiRNA2: AAGACGGCTGCCCGCAAGCCC; SiRNACon: AM4635-Ambion) Briefly, 24-well plates were seeded at 2.5 × 10^4 ^cells/well 24 h prior to transfection. Transfection mixes were set-up with 0.2 μg of siRNA in 50 μl of Optimem and 1 μl of lipofectamine in 50 μl of Optimem (per well). Transfection mixes were applied to the cells for 6 hours, subsequently removed and replaced with 1 ml of growth media. After 48 hours the cells were removed with trypsin and reseeded into 24-well plates and the transfection repeated as above. Seventy two hours after this second transfection the levels of TRPC6 protein was ascertained by SDS-PAGE electrophoresis and western blotting as described in the section above.

### Viability Assay

Cell viability was assayed using a trypan blue exclusion assay. Cells were removed with trypsin-EDTA and mixed with 0.4% trypan blue in PBS for 5 mins at room temperature. Subsequently the number of cells which were able to exclude the dye was counted.

### MTT assay

To six well dishes 600 μl of fresh medium and 150 μl of 5 mg/ml MTT [3-(4,5-dimethylthiazol-2-yl)-2,5-diphenyltetrazolium bromide] were added to the wells and incubated at 37°C for 2 h. The solution was removed and replaced by 890 μl of DMSO and 110 μl of Glycine buffer (0.1 M glycine, 0.1 M NaCl, pH 10.5) for 10 mins. The numbers of viable cells were then quantified by measuring the absorbance at 570 nm with a spectrophotometer.

### Data analysis

Data were analyzed by one-way ANOVA. Differences were taken as statistically significant when *P *< 0.05.

## Results

### Expression of TRPC mRNA in normal and breast cancer biopsy tissue

**Figure 1:**
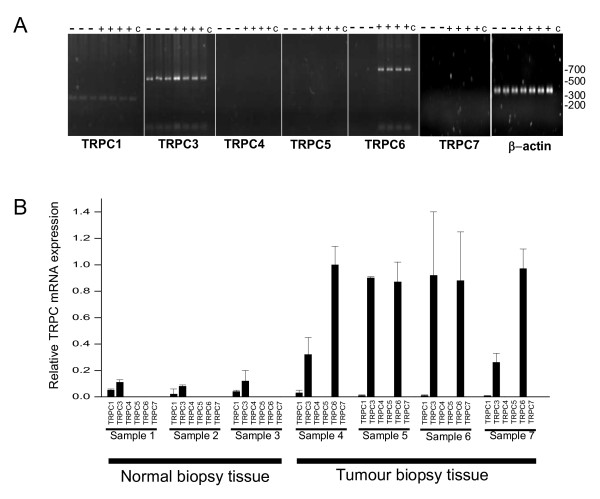
**Expression of TRPC mRNA in breast cancer biopsy tissue**. (A) PCR amplification with TRPC subtype specific primers of cDNA prepared from breast biopsy tissue (normal [-], tumour [+] and negative control [c]). The PCR reactions were separated on an agarose gel. Molecular weight markers in base pairs are shown to the right of the gels. As a control for the integrity of each cDNA sample β-actin was also analysed. (B) Relative expression of TRPC subtype specific mRNA in cDNA prepared from normal and tumour breast biopsy tissue. All data normalized to TRPC6 expression in sample 4.

Our aims were to determine which TRP channel sub-types are expressed in normal and cancer breast biopsy tissues. mRNA was prepared from seven breast tissue samples obtained by biopsy of which three were found to be normal and four were diagnosed as cancerous. The mRNA was converted to cDNA and TRPC sub-type specific primers (table 1) were utilized in a nested PCR design to confirm the expression of TRPC mRNA. Note that a nested PCR design was chosen since a single PCR amplification program (of 40 cycles) gave inconsistent results when attempted from breast tissues. Following PCR amplification all samples were separated on agarose gels and stained for DNA (Fig 1A). TRPC1 and TRPC3 were expressed in all samples; TRPC4, TRPC5 and TRPC7 expression was not observed in any of the samples; TRPC6 was expressed only in the cancer tissues. Note TRPC2 is a pseudogene in humans and was not tested. All primers were tested on known targets before use. Subsequently real-time PCR was utilized to compare the relative amounts of TRPC sub-types in the breast tissue biopsy samples (Fig 1B). Our results confirm that TRPC6 is not present in the normal breast tissue, while it is present in the cancer tissues (n = 5 for each sample). TRPC3 is present in normal tissues but it is significantly up regulated in the cancer tissues by an average of over five fold (F = 5.4 p = 2.5 n = 5). No significant up regulation of TRPC1 was found in the breast cancer tissues compared to the normal tissues (F = 5.3 p = 0.069 n = 5).

### Expression of TRPC mRNA in breast cancer epithelial cell lines

**Figure 2:**
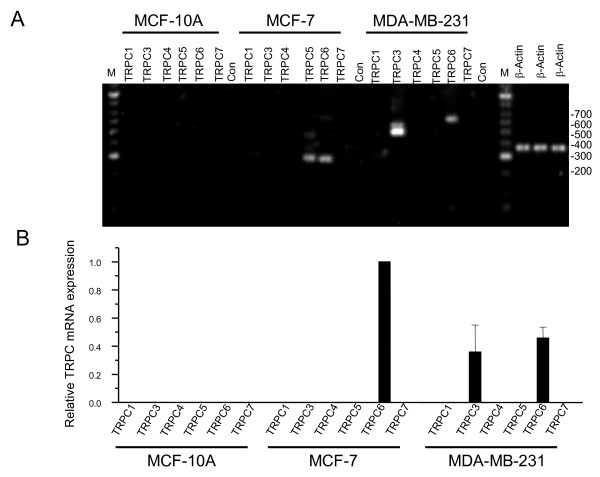
**Expression of TRPC mRNA in breast cancer epithelial cell lines**. (A) PCR with TRPC subtype specific primers was utilized to examine the expression of TRPC mRNAs in cDNA prepared from MCF-10A, MCF-7 and MDA-MB-231 cell lines. The PCR reactions were separated on an agarose gel. Molecular weight markers in base pairs are shown to the right of the gel. As a control for the integrity of each cDNA sample β-actin was also analysed. (B) Relative expression of TRPC subtype specific mRNAs in cDNA prepared from MCF-10A, MCF-7 and MDA-MB-231 cells. All data normalized to TRPC3 expression in MCF-7 cells.

Breast cell lines derived from cancer tissues are useful tools to understand the molecular factors involved in cancer and metastasis. In this study we chose three breast cell lines to investigate TRPC expression: MCF-10A (a non cancerous/transformed cell line), MCF-7 (lowly-metastatic cancer cell line) and MDA-MB-231 (highly-metastatic cancer cell line). mRNA was prepared from these cell lines and converted to cDNA. PCR amplification using TRPC sub-type specific primers was utilized and the PCR products were separated on agarose gels and visualized with a DNA stain (Fig 2A). PCR primers were designed to identify the existence of previously reported splice variants. For TRPC3 the main PCR products observed were larger in size (500 to 550 bp) to that expected (383 bp), this was found upon sequencing to correspond to a TRPC3β splice variant as reported previously [17]. For TRPC6 the PCR product found in the MDA-MB-231 cells was similar in size to that found in the cancer tissues from Fig 1. In the MCF-7 cells two splice-variants were visualized the full length expected PCR product (625 bp) and a predominant shorter splice variant (277 bp) these were subsequently sequenced and found to correspond to the splice variants (TRPC6 and TRPC6γ) as reported previously [14]. Real-time PCR was again utilized to compare the relative expression of TRPC sub-types in these three cell lines (Fig 2B). The results also shown in table 2 indicated that TRPC4 and TRPC7 are not expressed in any of the cell lines; a small amount of TRPC1 was found in all three cell lines; in MCF-7 cells a small amount of TRPC5 was present while the predominant TRPC subtype expressed was TRPC6. In MDA-MB-231 cells TRPC3 and TRPC6 were the predominant TRPC sub-types expressed. The difference between TRPC3 and TRPC6 expression in MDA-MB-231 cells was not significant at the 95% level (F = 0.22, p = 0.65 n = 5). Differences between TRPC6 expression in MCF-7 and MDA-MB-231 was significantly different at 95% level (F = 48.5, p = 4.4 n = 5) but this could be accounted for by consideration of the fact that MCF-7 cells are on average larger than MDA-MB-231 cells and therefore possess a larger plasma membrane [18].

**Table 2:** Relative expressions of TRPC channel sub-types in non-cancerous and cancerous breast cell lines

	**MCF-10A**	**MCF-7**	**MDA-MB-231**
**TRPC1**	2.3 × 10^-6 ^+/- 1.6 × 10^-6^	1.5 × 10^-7 ^+/- 3.9 × 10^-8^	5.0 × 10^-4 ^+/- 5.0 × 10^-4^

**TRPC3**	0	0	0.36 +/- 0.19

**TRPC4**	0	0	

**TRPC5**	0	0.0012 +/- 7.3 × 10^-4^	

**TRPC6**	0	1 (normalized)	0.46 +/- 0.08

**TRPC7**	0	0	0

### Protein expression and interaction of TRPC6 and TRPC3 in breast cancer epithelial cell lines

**Figure 3:**
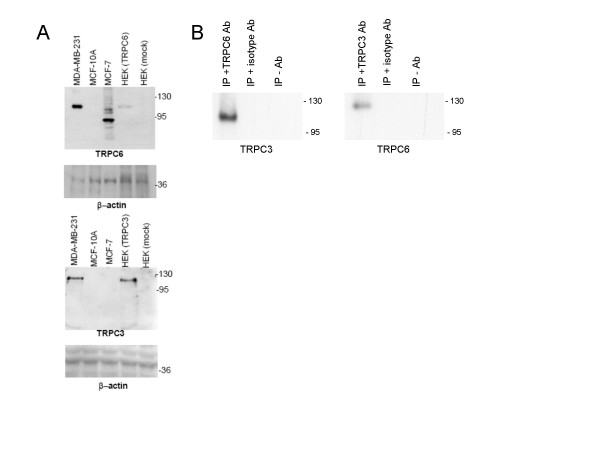
**Expression and immunoprecipitation of TRPC6 and TRPC3 protein in breast cancer epithelial cell lines**. (A) SDS-PAGE separation and Western blot analysis of protein extracts prepared from MCF-10A, MCF-7 and MDA-MB-231 cell lines. The blot was probed with a TRPC6 (top panel) or TRPC3 (lower panel) specific antibodies. As a TRPC6 or TRPC3 control protein extracts prepared from HEK cells transfected with a TRPC6 or TRPC3 expression plasmid and mock-transfected HEK cells were also separated. As a loading control the blots were also probed with an antibody to β-actin. (B) Immunopreciptation of TRPC6 and TRPC3 in MDA-MB-231 cells. Total protein extracts prepared from MDA-MB-231 cells were immunoprecipitated with specific antibodies to TRPC3 or TRPC6 or isotype antibody/no antibody as control. The total protein extracts and immunoprecipitations were separated on SDS-PAGE gels and Western blotted with antibodies to TRPC3 or TRPC6 (indicated below each blot). Sizes of marker proteins (kDa) are indicated to the right of all blots.

Extracts of protein were made from MCF-10A, MCF-7 and MDA-MB-231 breast cell lines and separated by SDS-PAGE. Following blotting to nitrocellulose the blots were probed with antibodies to TRPC6 (Fig 3A) and TRPC3 (Fig 3B). MCF-7 and MDA-MB-231 but not MCF-10A cells expressed TRPC6. As a positive control HEK cells transfected with a TRPC6 expression clone were also blotted. The results suggest that the MDA-MB-231 cells contained TRPC6 protein, which was close, or near the size of the full-length published clone, while the MCF-7 cells contained TRPC6 protein of a predominantly shorter form (possibly due to splicing observed in Fig 2). TRPC3 protein was observed only in the MDA-MB-231 cells and the size of this protein was close to the size observed from expression of the published sequence of TRPC3 in transfected HEK cells. Since the MDA-MB-231 cells had a similar amount of TRPC3 and TRPC6 mRNA expression we speculated that they might form a complex in these cells as they are reported to do so in other cells [19]. To confirm this we performed immunoprecipitation assays on protein extracted from MDA-MB-231 cells (Fig 3B) using antibodies to TRPC3 and TRPC6. Following SDS-PAGE and western blotting of the immunocomplexes the blots were probed with antibodies to TRPC3 or TRPC6. The results revealed that TRPC3 can immunoprecipitate TRPC6 and *visa versa*. No TRPC3/TRPC6 complexes were observed in the absence of an immunoprecipitating antibody or with isotype control antibodies.

### Localization and co-localization of TRPC6 and TRPC3 in breast cancer epithelial cell lines

**Figure 4:**
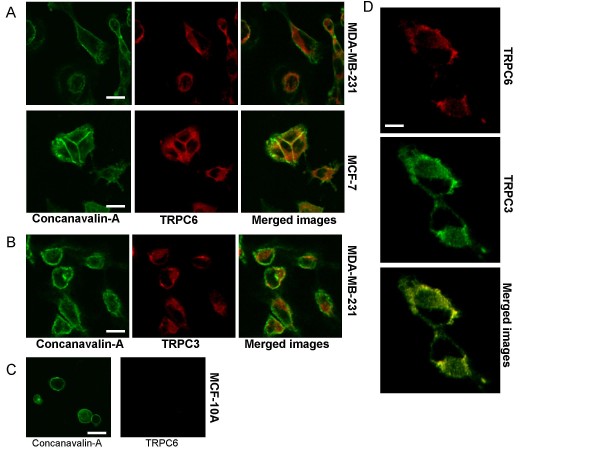
**Localization and co-localization of TRPC6 and TRPC3 in breast cancer epithelial cell lines**. (A) Immunoflourescent confocal images of TRPC6 protein expression in MCF-7 and MDA-MB-231 cell lines. Cells were treated with a concanavalin-FITC conjugate to stain the plasma membrane before permeabilization and probing with a TRPC6 specific antibody. Scale bar represents 20 μM. (B) Immunoflourescent confocal images of TRPC3 protein expression in MDA-MB-231 cells. Cells were treated with a concanavalin-FITC conjugate to stain the plasma membrane before permeabilization and probing with a TRPC3 specific antibody. The TRPC3/6 and conacavalin-FITC images were merged to indicate co-localization of the plasma membrane with TRPC3/6 expression. Scale bar represents 20 μM. (C) Immunoflourescent confocal images to determine TRPC6 protein expression in MCF-10A cells. Cells were treated with a concanavalin-FITC conjugate to stain the plasma membrane before permeabilization and probing with a TRPC6 specific antibody. Scale bar represents 40 μM. (D) Co-localization of TRPC6 and TRPC3 in MDA-MB-231 cells. Images were merged to indicate co-localization of TRPC3 and TRPC6 antibody staining. Scale bar represents 32 μM.

Our PCR and protein results suggested that TRPC6 and/or TRPC3 were the predominant TRPC sub-types expressed as mRNA in the MCF-7 and MDA-MB-231 cell lines. To identify the localization of these TRPC channels in these cell lines we utilized immunochemistry staining with TRPC6 and TRPC3 specific antibodies. The MCF-7 and MDA-MB-231 cells were fixed and subsequently stained with a concanavalin-FITC conjugate. Following permeabilization the cells were probed with antibodies specific to TRPC6 or TRPC3 and visualized by confocal microscopy (Fig 4A, n = 4). TRPC6 was expressed in MCF-7 and MDA-MB-231 cells but only a small fraction of protein (10 and 3% respectively) was expressed on the plasma membrane. TRPC3 was expressed in MDA-MB-231 cells (Fig 4B, not tested in MCF-7 or MCF-10A cells) and the localization was predominantly intracellular with 4% of protein being expressed on the plasma membrane. MCF-10A cells did not express TRPC6 (Fig 4C). Co-staining of MDA-MB-231 cells with TRPC3 and TRPC6 antibodies revealed a similar co-localization (Fig 4D).

### Effect of a TRPC6 activator and gene silencing of TRPC6 on growth of breast epithelial cell lines

**Figure 5:**
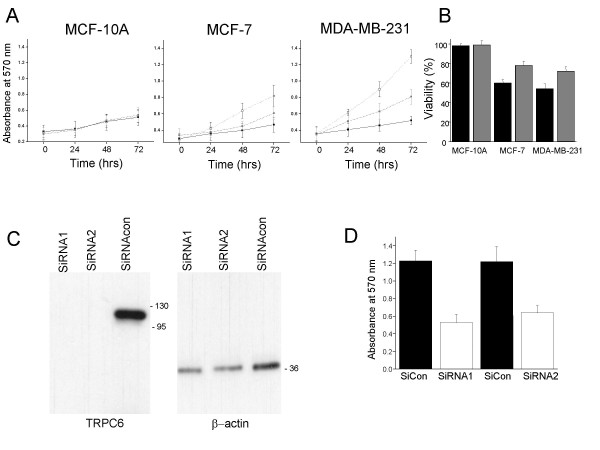
**Effect of TRPC6 activators and gene silencing of TRPC6 on growth of breast cancer epithelial cell lines**. (A) MCF-10A, MCF-7 and MDA-MB-231 cell lines were seeded into six-well plates and incubated for 24 hours at 37°C. Subsequently, hyperforin was added at a concentration of 10 and 1 μM and the cells were incubated at 37°C. As controls, cells were also mock-treated with DMSO (which was used to dissolve the hyperforin). At 0, 24, 48 and 72 hours post incubation cell growth was measured using a MTT assay (black square 10 μM, grey square 1 μM hyperforin and empty square mock-treated). (B) MCF-10A, MCF-7 and MDA-MB-231 cell lines were seeded into six-well plates and incubated for 24 hours at 37°C. Subsequently, hyperforin was added at a concentration of 10 and 1 μM and the cells were incubated at 37°C. As controls, cells were also mock-treated with DMSO (which was used to dissolve the hyperforin). At 72 hours post incubation viability was measured and normalized against the mock-infected cells (black square 10 μM and grey square 1 μM hyperforin). (C) TRPC6 in MDA-MB-231 cells was silenced using a SiRNA double transfection method. At 72 hours post the second transfection the levels of TRPC6 was ascertained by protein extraction and Western blotting. As loading controls the lower part of the gel was removed and probed with an antibody to β-actin. Two SiRNAs were utilized and compared to a SiRNA control. (D) TRPC6 in MDA-MB-231 cells was silenced using siRNA transfection. At 72 hours post the second transfection cell growth was ascertained using a MTT assay. Two SiRNAs were utilized and compared to a SiRNA control.

Hyperforin was reported as a specific activator of TRPC6 compared to other TRPC channels [20]. Since TRPC6 was up regulated in MCF-7 and MDA-MB-231 cells compared to MCF-10A cells we decided to investigate the effect of this lipophillic compound on cell growth. The results (Fig 5A) indicated that for the MCF-10A cell line no significant change in the growth occurred when hyperforin was applied for 24, 48 or 72 hrs at a concentration of 10 μm or 1 μm (F = 0.23 p = 0.68 n = 5 for 72 hrs). In contrast (Fig 5A) for the MCF-7 and MDA-MB-231 cells a significant reduction in cell growth was noted (for 72 hrs F = 31 p = 0.010 n = 5; F = 33 p = 0.006 n = 5 respectively). No significant effect was seen with hyperforin at concentrations of 0.1 μm or lower for all three cell lines (data not shown). The effect of hyperforin on viability was also ascertained at 72 hrs post addition at concentrations of 10 μm and 1 μm (Fig 5B). No significant effect was observed in the MCF-10A cell line at 1 μM (F = 0.11 p = 0.68 n = 5), but a significant effect was seen for MCF-7 and MDA-MB-231 cell lines at 1 μm (F = 39 p = 0.001 n = 5; F = 56 p = 0.0005 n = 5). A double SiRNA transfection protocol was used to ensure complete knockdown of TRPC6 in MDA-MB-231 cell lines (Fig 5C). Two independent SiRNA's were transfected and their effects on cell growth was ascertained using an MTT assay (Fig 5D). The results indicate a significant effect on cell growth at 72 hrs post transfection compared to cells transfected with a control SiRNA control (F = 102 p = 0.002 n = 5; F = 87 p=0.004 n = 5).

## Discussion

The ability of Ca^2+ ^signalling to regulate pathways such as proliferation and apoptosis suggests that therapies that modulate Ca^2+ ^signalling in cancer cells might be a therapeutic option [5]. Modulating the activity of Ca^2+ ^channels and pumps that are aberrantly expressed in cancer cells might sufficiently disrupt Ca^2+ ^homeostasis to selectively target cancer cells [5]. The primary question to ask is which Ca^2+^channels are expressed in cancer cells? To date there is incomplete information as to the expression of TRP channels in normal and cancer breast tissues. Our major conclusions from this research are listed below:

1. Our experiments suggested that normal breast tissues expressed the TRPC1 and TRPC3 genes. Comparison of normal and breast cancer biopsy tissues indicated a significant alteration in TRPC channel expression both in terms of channel type expressed and levels of channel expression. In breast cancer biopsy tissues TRPC6 was expressed and appeared to be the predominant TRPC channel expressed. Additionally TRPC3 appeared to be significantly up regulated in breast cancer tissues. Of course our experiments could not distinguish which cell types these TRPC channels were expressed in, since the breast tissue could contain a variety of cell types.

2. In the epithelial non-cancerous cell line MCF-10A only relatively very low amounts of TRPC1 expression was observed. In lowly metastatic breast cancer cell line MCF-7 the predominant channel expressed was a short splice variant of TRPC6. In the highly metastatic breast cancer cell line MDA-MB-231 the predominant TRPC channels expressed were TRPC3 and TRPC6 (of longer splice form variants). The amounts of TRPC3 and TRPC6 expressed in the MDA-MB-231 cell line were comparable in level, which leads us to speculate that they may form heteromultimers. The sizes of PCR products for TRPC3 and C6 found in the cancer tissues appeared to be consistent to that found in the MDA-MB-231 cell line.

3. Protein expression of TRPC3 and TRPC6 in breast cancer cell lines appeared to be consistent with the PCR results. TRPC6 and TRPC3 expression was observed in the MDA-MB-231 cell line with the majority of protein being expressed in an intracellular location.

4. Our data provides strong evidence that TRPC3 and TRPC6 form heteromutimeric channels in breast cancer epithelial cell line MDA-MB-231, which is consistent with previous reports of TRPC3/6 expression [19].

5. Hyperforin a specific activator of TRPC6 produced a significant reduction in cell growth for the breast cancer cell lines, while the non-cancerous breast cell line was unaffected. Viability was significantly reduced in the breast cancer cell lines. TRPC6 activation is known to promote cell proliferation. This is an opposite result which is obtained here when using hyperforin however it may be expected that overactivation of TRPC6 by hyperforin could cause a disruption in Ca^2+ ^signalling hence affecting cell proliferation. In an earlier study, hyperforin induced apoptosis and inhibited the growth of various human and rat tumour cell lines *in vivo*, [21]. Our results provide preliminary functional data that a rationale to target TRPC channels in breast cancer cells may have some promise.

6. Complete silencing of TRPC6 in MDA-MB-231 cells using transfected SiRNA resulted in a significant reduction in cell growth but not viability.

On the whole our results with the MCF-7 cell line confer with the recent report by Bolanz *et al *[22] who found that TRPC6 and TRPV6 are the predominant TRP channels expressed in the lowly metastatic cell line T47D. Interestingly our data suggests that in the highly metastatic cell line MDA-MB-231 TRPV6 was down regulated and TRPC3 was up regulated. Our results are also consistent with the previous report by El Hiani *et al *[13] who suggested that, in MCF-7 cells, TRPC1 and TRPC6 are the most likely candidates for the highly selective Ca^2+ ^current described in these cells. We propose that TRPC6 is the major TRPC channel expressed in MCF-7 cells. However correlation of the permeation and pharmacological profiles of the cationic current in MCF-7 cells as described by El Hiani *et al*, [13] with those described for TRPC6 shows a number of notable differences. The authors of that paper suggest that the cationic channels in MCF-7 cells are probably heteromultimers that include both TRPC6 and TRPC1 together with some other TRP members. Given the presence of TRPV6 and TRPC6 in MCF-7 cells we support this view. More recently Guilbert *et al *[14] demonstrated that TRPC6 was expressed and functional in breast cancer epithelial cells and that this channel was overexpressed in tumour tissues without any correlation with tumour grade, ER expression and lymph node metastasis. TRPC proteins constitute a family of conserved Ca^2+^-permeable cation channels which are activated in response to agonist-stimulated PIP(2) hydrolysis and subsequent studies have provided substantial evidence that some TRPCs contribute to SOC activity [7]. Since Ca^2+ ^is known to be involved in a number of metastatic processes including motility and proliferation [5] these processes may result in store-depletion thus up regulation of genes which contribute to SOC activity may plausibly be necessary for these processes to take place efficiently.

## Competing interests

The authors declare that they have no competing interests.

## Authors' contributions

EA contributed to the siRNA and growth curves and helped to draft the manuscript. MD participated in its design and coordination. SK performed the PCR and immunoflurescence. CPP performed the remainder of assays conceived and participated in its design and coordination and drafted the manuscript.
